# Views of professionals and volunteers in palliative care on patient-centred care: a Q-methodology study in the Netherlands

**DOI:** 10.1186/s12904-019-0479-5

**Published:** 2019-11-08

**Authors:** Milanne M. J. Galekop, Hanna M. van Dijk, Job van Exel, Jane M. Cramm

**Affiliations:** Erasmus School of Health Policy and Management, Erasmus University Rotterdam, Burgemeester Oudlaan 50, 3000 DR Rotterdam, The Netherlands

## Abstract

**Background:**

Patients with palliative care needs, require support with their physical needs, but also with their emotional, spiritual and social needs. Patient-Centred Care (PCC) may help organizations to support these patients according to their needs and so improve the quality of care. PCC has been shown to consist of eight dimensions, including for instance access to care and continuity of care, but these eight dimensions may not be equally important in all care settings and to all patients. Furthermore, the views of those involved in care provision may affect the choices they make concerning care and support to patients. Therefore, insight into how professionals and volunteers involved in palliative care delivery view PCC is important for understanding and improving the quality of care in the palliative sector.

**Methods:**

This study was conducted in the palliative care setting (hospices and hospitals) in the Netherlands. Views on palliative care were investigated using the Q-methodology. Participants were asked to rank 35 statements that represented the eight dimensions of PCC in palliative care settings, and to explain their ranking during a follow-up interview. Ranking data were analysed using by-person factor analysis. Interview materials were used to help interpret the resulting factors.

**Results:**

The analysis revealed two distinct viewpoints on PCC in palliative care: ‘The patient in the driver seat’, particularly emphasizing the importance of patient autonomy during the last phase of life, and ‘The patient in the passenger seat’, focussed on the value of coordination between professionals, volunteers and patients.

**Conclusions:**

The most distinguishing aspect between views on PCC in palliative care concerned control; a preference for the patient in the driver’s seat versus shared decision-making by a team consisting of patient, professionals and volunteers. Different types of care and support may be most adequate to satisfy the different needs and preferences of patients with either of these views.

## Background

Patients with palliative care needs, require support with their physical needs, but also with their emotional, spiritual and social needs [1, 2]. Patient-Centred Care (PCC) may help organizations to improve the quality of care delivery by supporting patients in the last phase of their life with their needs, according to their own preferences. PCC is defined as “providing care that is respectful of and responsive to individual preferences, needs and values and ensuring that patient values guide all clinical decisions [3].” Such care is expected to be especially beneficial to patients with palliative care needs. A study by Dy and colleagues [4] systematically reviewed the evidence on palliative and health care interventions that aimed to improved outcomes for patients with advanced and serious illness, and found the strongest evidence of effectiveness in interventions that incorporated patient-centred quality improvement components, such as family, patient or caregiver education and self-management. This supports the importance of PCC in palliative care. Eight dimensions have been identified to be important to PCC: respect for patients’ values, preferences and expressed needs; provision of information and education; access to care; emotional support to relieve fear and anxiety; involvement of family and friends; continuity and secure transition between healthcare settings; physical comfort; and coordination of care [5, 6]. Research showed the benefits of investing in these eight dimensions for health care in general [7] as well as for the palliative care setting more specifically [1, 2]. However, previous studies have shown that views on the relative importance of these eight dimensions for PCC may differ between professionals [8, 9]. Understanding such differences is important because different views on PCC may translate into different priorities for care delivery. These previous studies were conducted in a hospital setting, but little is known about views on PCC in the palliative care setting.

Palliative care is given in regular settings, like hospitals and nursing homes, and in specialized palliative care centres, also called hospices [10]. In 2011, 45% of all deaths in the United States –concerning approx. One million people- were users of hospices [11]. Moreover, by 2012, there were 5500 hospice programmes in the United Kingdom, which reflects a steady increase since the first opening of such a programme in 1974. In the Netherlands, the use of hospices has also increased considerably. In 2012, about a quarter of hospitals in the Netherlands had beds or day treatment facilities for palliative patients. At the same time, there were 192 hospices across the country, which by their number and regional dispersion have an important role in providing care and support in the palliative care setting [10].

Given that professionals play a central role in delivering PCC [12, 13] understanding their views on PCC is essential for achieving further improvement of quality of care in the palliative care setting. Therefore, this study aims to explore their views on PCC. Moreover, volunteers are an integral part of palliative care in the Netherlands, especially in hospices [14]. They work together but are also complemental to professionals. In total, more than 10,000 volunteers provide help to patients with palliative care needs at home and in hospices [15]. Because of their important role in the palliative sector, additional aims of this study are to explore the views of these volunteers on PCC as well, and to see whether their views align with or differ from those of the professionals.

## Methods

### Design

Q-methodology was used to identify views of professionals and volunteers on PCC. This method combines quantitative and qualitative techniques to study subjective phenomena in a systematic way [16]. Typically, respondents are asked to rank a set of statements of opinion on the topic of study according to agreement or importance and to explain their ranking [17]. Recently, Q-methodology was for example used to study perceptions of general practitioners (GPs) of their role in palliative care for children [18], and to study views on PCC among professionals in a New York hospital [8] and nephrology patients in a hospital in the Netherlands [9].

### Setting and participants

To ensure a wide representation of views, this study was performed in two hospitals and six hospices in the Netherlands (Table 1). Four of the six hospices were of Christian denomination, one hospital was an academic medical centre. Interviews were conducted with 41 respondents, consisting of 30 professionals and 11 volunteers. Considering that this was a first explorative study of this topic in the palliative care setting and the potential burden of the interview, both in terms of content and duration, patients with palliative care needs were not included in the current study.

**Table 1:** Sample

Institution	Function	N
1. Academic hospital	Radiotherapist	1
	Gastroenterologist	1
	Radiotherapist (AIOS)	2
	Spiritual caregiver	1
2. Hospital	Doctor palliative medicine	1
	Nurse specialist PC	2
	Nurse Surgery	1
	Nurse Oncology	2
	Nurse CCU	1
	Nurse Dialyse	1
	Nurse Geriatrics	1
	Spiritual caregiver	1
3. General (Christian) hospice with 6 rooms	Nurse	1
	Volunteer	4
4. General hospice with 4 rooms	Nurse	1
5. General (Christian) hospice with 7 rooms	Nurse	3
6. General hospice with 4 rooms	Nurse and coordinator	1
	Nurse	1
	Nurse (in Training)	1
7. General (Christian) hospice with 8 rooms	Specialist geriatrics	1
8. General (Christian) hospice with 6 rooms	Specialist geriatrics	1
	Nurse and coordinator	1
	Nurse	4
	Volunteer	7

### Statements

The statement set used in this study was based on two previous studies into views on PCC [8, 9]. These statements covered the eight dimensions of PCC developed by the Picker Institute [5, 6]. Based on inspection of previous literature on this topic in the context of palliative care [10, 11, 14], a number of adaptations were made to the statement set. These adaptations were made together with a nurse specialist palliative care. Next, we conducted three pilot interviews with members of the target population in order to assess the comprehensiveness and intelligibility of the statement set. Based on the comments collected during these pilot interviews, we decided to add two statements to the set, namely statement 6. Healthcare professionals pay attention to the spiritual and psychosocial needs of patients and statement 23. Low cognitive functioning (for example: dementia) is not a barrier for receiving good quality of care. Moreover, we decided to delete two statements that were considered redundant, namely ‘clear directions are provided to and inside the hospital’ and ‘patients receive skilled advice about care and support at home after hospital discharge’, since these are less applicable to patients with palliative care needs. Some small adaptations in a few other statements were done and this resulted in a final set of 35 statements. See Table 3 in Appendix for a complete overview of the adaptations made to the statement set adopted from two previous studies [8, 9].

### Data collection

**Fig. 1:**
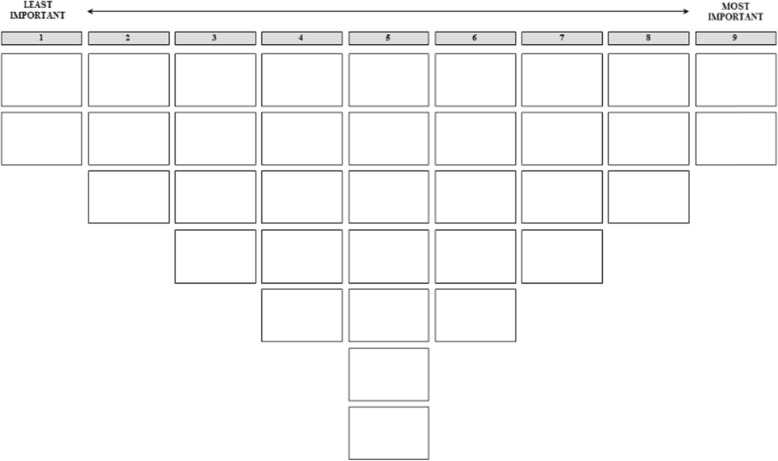
Sorting grid

Interviews were conducted at the working place of participants, i.e. in the different hospitals and hospices. In the case of the hospices, coordinators of the hospices (spokesperson and in charge of organizing and coordinating the care with patient and family) were approached via email or telephone and asked if their organization would be willing to participate in this study. If so, the coordinator then scheduled interviews with professionals and volunteers working in their hospice during working hours. Six of the eight randomly selected hospices in two regions in the Netherlands that were approached agreed to participate in the study; one declined, and one did not respond. In the case of the hospitals, the professionals were approached directly via email or telephone, and interviewed were scheduled with them personally. Professionals at two hospitals in the same two regions were approached. Interviews were conducted by one researcher and lasted approximately 50 min. Informed consent was asked at the beginning of the interview and no relation existed between the participants and researchers. The interviews were recorded and transcribed, after permission was obtained from the respondent, whereby the anonymity and confidentiality of the respondent was ensured. During the interview, respondents were asked to a) rank the statements according to their perceived importance for PCC using a sorting grid ranging from 1 (least important) to 9 (most important) (Fig. 1) and b) elaborate on their ranking, paying most attention to the statements placed in the outer columns.

### Analyses

A by-person factor analysis was done to identify clusters in the ranking data using PQMethod2.11 [19]. For each identified factor a weighted average ranking of the statements was computed, and interpreted and described as distinct views on PCC. Distinguishing statements (ranked significantly different in a factor compared to the other factors) and consensus statements (ranked similarly across factors) were identified. Respondents’ explanations of their ranking were used to verify the interpretations, and some quotes selected to illustrate the views in respondents’ words. Furthermore, possible differences in views between professionals and volunteers were inspected using the factor associations of respondents.

## Results

The majority (87%) of respondents were women and the age of respondents ranged from 30 to 68 years, with an average age of 52 years. The factor analysis revealed two distinct views on PCC, explaining 40% of the variance. Both viewpoints are supported by professionals and volunteers. The statement scores in each factor are shown in Table 2. Of the 35 statements included in the set, 20 statements were ranked statistically significantly differently (*p* < 0.01) between the two factors; the remaining 15 statements were ranked similarly in the two factors.

**Table 2:** Rank scores of statements for views on patient-centred care

Dimensions of PCC	Statements	View 1	View 2
*Patients’ preferences*	1. Healthcare professionals treat patients with dignity and respect.†	+ 4	+ 4
	2. Healthcare is focused on improving the quality of life of patients.†	+3	+ 4
	3. Healthcare professionals consider patient preferences.†	+ 2	+ 1
	4. Healthcare professionals involve patients in decisions regarding their care.	+ 2*	+3
	5. Patients are supported to set and achieve their own goals.†	0	0
	6. Healthcare professionals pay attention to the spiritual and psychosocial needs of patients.†	+ 2	+ 2
*Physical comfort*	7. Healthcare professionals pay attention to pain management.†	+3	+3
	8. Healthcare professionals take patient preferences for support with their daily living needs into account.	+ 2*	0
	9. Patient areas are clean and comfortable.†	−3	-3
	10. Patients have privacy.	+ 1*	+ 2
*Coordination of care*	11. Healthcare professionals are well-informed; patients need to tell their story only once.	0*	-3
	12. Patient care is well-coordinated between professionals.	+ 1*	+ 2
	13. Patients know who is coordinating their care.	−4*	−2
	14. Patients have a first point of contact who knows everything about their condition and treatment.†	−3	−3
	15. Healthcare professionals work as a team in care delivery to patients.	−1*	+ 1
*Emotional support*	16. Healthcare professionals pay attention to patients’ anxiety about their situation.	+ 1*	+ 2
	17. Healthcare professionals involve relatives in the emotional support of the patient.†	0	0
	18. Healthcare professionals pay attention to patients’ anxiety over the impact of their illness on their loved ones.	0*	+ 1
*Access to care*	19. The building is accessible for all patients.	−2*	−1
	20. It is easy to schedule a conversation with a doctor or nurse.	−1*	+ 1
	21. Waiting times for a request of a patient (for example: a treatment, medication or food) is acceptable.†	−1	0
	22. Language is not a barrier for access to qualitative good care.	−3*	−1
	23. Low cognitive functioning (for example: dementia) is not a barrier for receiving good quality of care.	−1*	0
*Continuity and transition*	24. When a patient is transferred to another ward, relevant patient information is transferred as well.†	−2	−2
	25. Patients who are transferred are well-informed about where they are going, what care they will receive and who will be their contact person.†	−2	− 2
*Information and education*	26. Patients are well-informed about all aspects of their care.	+ 1*	0
	27. Patients can access their care records.	−4*	−4
	28. Patients are in charge of their own care.	+ 4*	−2
	29. Healthcare professionals support patients to be in charge of their care.	+3*	−1
	30. There is open communication between patient and healthcare professionals.	+ 1*	+3
	31. Healthcare professionals have good communication skills.	0*	+ 1
*Family and friends*	32. Accommodation for relatives is provided.†	−2	−4
	33. Healthcare professionals involve relatives in decisions regarding the patient’s care.†	−1	0
	34. Healthcare professionals pay attention to loved ones in their role as carer for the patient.	0*	−1
	35. Healthcare professionals pay attention to the needs of family and friends of the patient.†	0	−1

Distinguishing statements: * P < 0.01

*Consensus statements:* †

*Scores range between − 4 and + 4 and correspond to the columns of the sorting grid (see* Fig. 1*): − 4 concerns “least important”; + 4 concerns “most important”*

### Viewpoint 1: ‘The patient in the driver seat’

Respondents holding this view strongly believe that patients should be in charge of their own care and that professionals and volunteers should primarily support patients to achieve their goals (28;+ 4* (statement 28;score + 4*), 29;+ 3*). Participants strongly feel healthcare professionals and volunteers should respect patients’ autonomy; *‘Well, I believe that patient autonomy is a priority and we adjust the care we provide accordingly. This means that one can sympathize with others, that ‘nothing is set’ and everything is well communicated, and that the patient has sovereignty. Sovereignty… well... actually more like autonomy’* (respondent 1). Respondents further explained that care should be provided keeping patients’ preferences in mind (3;+ 2, 8;+ 2*): *‘I think the patient needs to express what he or she wants and the care will then be provided according to his or her needs’* (respondent 5). Those holding this viewpoint feel that patients should not only be in control when it concerns care-related aspects, but in all other areas of life as well; *‘There are patients, for example, who don’t want any help, who prefer to sit (alone) in their room, patients who have their own coffee machine, or patients who turn around their day-night routine, all of this is good’* (respondent 13), *‘They are in charge of their body, their lives and their care’* (respondent 30).

Patients lose a lot during this last phase of their life, so it is important to support them even in the small things they cling to, ensuring their sense of control (29;+ 3*) *‘Most importantly, patients should feel like they have the freedom to act independently; that they are autonomous, despite that so much is happening around them that they have no control of. You [as a caregiver/ professional] try, as much as possible, to give them the feeling of control in every aspect of life’* (respondent 12). According to people holding this viewpoint this requires professionals that are sensitive to patient’s (changing) needs: *‘As a professional you have to be there for the patient, but sometimes you also have to give some space to the patient. You shouldn’t take a leadership role, or present yourself as dominant figure, sometimes you need to take a step back’* (respondent 14). And because control is in hands of the patients instead of the professionals or volunteers, it is less important that the patient knows who coordinates his or her care (13;-4); ‘*Understanding this (the care process) is not important for good quality of care, it is simply just not a priority’* (respondent 33). It is also considered to be less important that healthcare professionals work as a team (15;-1*) and that the care is well-coordinated between professionals (12;+ 1*), since patients ‘lead’ the team. Respondents holding this viewpoint do state the importance of alignment of goals between patients and professionals: *‘Professionals and the patient have a mutual goal, and how this mutual goal is reached is less of importance’* (respondent 30).

### Viewpoint 2: ‘The patient in the passenger seat’

In contrast with viewpoint 1, those holding this second viewpoint considered it most important that patients, volunteers and professionals work together as a team with *the patient in the passenger seat*. They feel that shared decision-making is important in all aspects of care, whether it concerns taking a shower, or decisions about medication or treatment. Whenever possible patients make their own choices, often after consultation with the professional. But when they are not willing or capable to decide themselves at any stage of their care, for example because they lack the energy or capacity to be involved, the professional needs to step in and decide on their behalf, in their interest. This is in contrast with professionals and volunteers with viewpoint 1, who think it is important that patients are always in charge of their own care. Professionals and volunteers in this viewpoint in a way thus play a central role in the decision-making process in this viewpoint and according to them patients are fine with that (28;-2, 29;-1); *‘In the last phase of life [being in charge of their own care] is not necessary anymore. I think patients are allowed to expect that everything is going alright’* (respondent 29), *‘People who are in the last phase of their life often say: “you can make the decisions, I’m tired of doing that. If you make good decisions, I can just ‘be’ ill. Spare me all the choices”’* (respondent 6). Respondents holding this viewpoint, in contrast with viewpoint 1, further explained that patients do want to talk about their care but find it hard to be in charge of their own care, make the (tough) decisions and to have the last saying; ‘*They (patients) don’t know what the different possibilities are or even what they want, and therefore it is important that the professionals and volunteers who know the different possibilities, discuss them with the patients as much as possible”* (respondent 18).

For care-related decisions it thus remains important that patients are involved (4;+ 3). However, in this viewpoint it is seen as important that professionals, volunteers and patients should team-up and take a decision collaboratively, or at the least, professionals and volunteers should inform patients. Open communication helps to achieve these goals (30;+ 3). *‘As a professional or volunteer, you try to be as open as possible. Not necessarily about their illness, but rather about understanding what a patient wants and whether something is painful or not’* (respondent 28). If open communication becomes the norm, patient’s access to their medical record becomes less important (27;-4); *‘To have access to their medical files is not necessary when you provide good quality care’* (respondent 16), *‘We explain everything thoroughly, they won’t require access to their medical files’* (respondent17).

Finally, those holding this view indicated that according to their experience patients *do* want to tell their story several times, because it is part of their acceptation process and thus positively affects their well-being (11;− 3). *‘Them telling their story helps them understand what is going and gives some relieve, this is also sometimes the beginning of acceptance’* (respondent 21).

The professional, volunteer and patient act together, to achieve the best possible care. Mutual trust between the patient, professionals and volunteers seems to be a prerequisite, to get to know what good quality of care for a patient is and act upon that.

### Consensus between views

Although both viewpoints ranked most of the statements differently, 15 statements were ranked similarly (see Table 2: †), with three of these statements ranked as highly important for PCC: ‘dignity and respect’ (view 1: 1;+ 4 (statement 1, ranked at place + 4), view 2: 1;+ 4) ‘quality of life’ (view 1: 2;+ 3, view 2: 1;+ 4) and ‘pain management’ (view 1: 7;+ 3, view 2: 7;+ 3). Professionals and volunteers consider dignity and respect and quality of life as the foundation of good care-delivery; *‘People are different, some even rude but they all deserve to be treated with dignity’* (respondent 29); ‘*In my opinion, (in the end) it’s all about well-being given that well-being encompasses almost everything’* (respondent 32). Respondents also see pain management as a requirement for good care; *‘It is simply the first priority in terms of treatment here at this hospice, to make someone (patients) feel comfortable’* (respondent 13). All other aspects of care are considered inferior. For example; the statement ‘patient areas are clean and comfortable’ (9), scoring -3 in both views, is found less important for PCC according to both views. One important reason for this is that respondents prefer to give more attention to the patients themselves rather than to their rooms. Following the argument of the respondents, it is also what patients themselves consider less important; ‘*I was cleaning a room of one patient, but then another patient wanted to talk with me. In these cases, giving your attention to the patient is far more important than cleaning. Giving attention to patients who need it always has the priority over anything else*’ (respondent 26). Respondents also see ‘clean’ and ‘comfortable’ as self-evident things in health care; *‘Which hospital doesn’t clean up its own mess? I think this is something you always do and should be obvious. Just like it’s logical to have the opportunity to have family sleep over, to be close to the patient, when the patient is in a terminal phase of their illness’* (view 1: 32;-2, view 2: 32;-4) (respondent 21). At the same time, having an accommodation for family is considered less important. Respondents in both views state that it is important to attend to the preferences of patients first, and to those of the family thereafter. In general, there is consensus about the role of the family of patients in decision-making (statements 32, 33 and 35); *‘Of course there are some meetings involving the whole family, but ultimately, it is the patient who decides and not the family’* (respondent 13).

Finally, the statements from the continuity and transition domain (24, 25) receive scores of − 2 in both views. This might be explained by the fact that patients are usually not transferred during this last stage of their life. Especially in a hospice this happens only rarely; *‘Patients usually pass away here; it is very rare that the patients transfer somewhere else from our facility’* (respondent 27).

## Discussion

This study explored the relative importance of the eight dimensions of PCC in the palliative care setting among professionals and volunteers. Two main viewpoints were identified. Respondents representing viewpoint 1 ‘The patient in the driver seat’, find it important that patients keep their autonomy during the last phase of life. According to them patients should be in charge of their own care and professionals and volunteers should act according to the preferences of patients. This contrasts with the viewpoint 2, ‘The patient in the passenger seat’, where PCC is found to be best delivered when professionals, volunteers and patients *share* control with the patient in the passenger seat. Earlier research confirms that while some patients who receive palliative care want to actively take part in the decision-making process others prefer decisions are made for them [20].

While in some aspects the two viewpoints differed, we also found several agreements. Both viewpoints ranked the statements concerning patient preferences as highly important; especially the statements about quality of life, and dignity and respect. These aspects were seen as the foundation of PCC delivery in the palliative care setting. But, as dignity is something which is subjectively experienced by patients and each patient is unique in their requirements, as a professional it is important to adopt an open approach and learn to know from each patient how they think about dignity [21]. This open approach consists of verbal and non-verbal communication, between patients, volunteers and professionals. In this study we found that open communication was ranked relatively high in both views. Earlier research [22–24] supports these findings, showing that communication should always be ‘open’ and based on ‘trust’. Shannon and colleagues [25] argued that effective communication indeed is very important around end-of-life care, because symptom control and pain management is impossible without effective communication [26]. In other words, open communication is imperative for professionals and volunteers in palliative care in order to truly understand the needs, values and perspectives of patients, and thus crucial when providing care to patients with dignity and respect.

Access to the care records is considered less important in PCC care-delivery for patients receiving palliative care. According to the respondents, medical care records are often far too complex for patients and sometimes even cause fear. Moreover, they state that if patients want information, it is better told face-to-face. This contrasts the results of a systematic review [27] showing that access to medical records appeared to enhance patients’ perceptions of control and reduced or had no effect on patient anxiety. These studies, however, did not include patients with palliative care needs and/or end-of life care, which may explain the difference in findings. When you are nearing the end of your life, ‘technical’ medical records may become less important, compared to patients who are dealing with an acute or chronic condition that is not immediately life-threatening.

A remarkable finding of this study is that respondents holding viewpoint 2 found it less important that patients should only need to tell their story once. They argued that it is important for patients in the palliative phase to tell their story repeatedly, as this helps accepting the idea of imminent death [28, 29]. Other research confirms the notion that patients in hospices have to deal with their pain, including their physical, spiritual, psychological and social pain, by talking about it until the end [30]. While telling your medical ‘facts’ over and over again may be experienced as annoying or even cumbersome, telling your *life story* and *pain* may help dealing with the process of dying.

Two previous studies used the same method and largely the same statement set as used in this study. Berghout et al. [8] investigated the views of professionals in hospitals on patient-centred care, and identified three views: “treating patients with dignity and respect,” “an interdisciplinary approach” and “equal access and good outcomes.” It may not be surprising that these views differ from the views we found in this study, since the setting of the study is clearly different. An important similarity is that professionals in both settings find treating patients with dignity and respect an important aspect of PCC. Cramm et al. [9] explored the views of patients with end-stage renal disease receiving dialyses and healthcare professionals working at a haemodialysis department. They found four views on what is important for PCC in end-stage renal disease, that focused on: “listening to patients and taking account of their preferences in treatment decisions,” “providing comprehensible information and education to patients so that they can take charge of their own care,” “the atmosphere at the department” and “having a professional or acquaintance that acts as care coordinator, making treatment decisions with or for patients”. Although these views again differ somewhat from the ones found in our study, there also are important similarities. One viewpoint also stated that it is important that patients are in charge of their own care, while two other viewpoints express different notions of shared decision-making. Also in this study, involving the family is considered less important for PCC.

Lastly, this study comes with some limitations. Although the statement set was developed carefully, some issues should be mentioned. Statement 22 ‘*Language is not a barrier for access to qualitatively good care*’ was sometimes regarded difficult to understand, because of the formulation, and consequently also difficult to rank. Secondly, trust appeared to be an important issue during the interviews. In line with previous research [31], patients’ trust in professionals is one of the most important elements of palliative care. There was no specific statement about ‘trust’ in this study, but it may be good to add such a statement in future research in this area. The same applies to ‘care after death’ for family and friends. In our study the dimension ‘family and friends’ was not ranked very highly, but caring for family and friends after death seemed to be an important aspect in this dimension that was missing in the statement set [32]. Thirdly, the composition of the respondents may be somewhat biased; only five men participated as compared to 36 women. Although it is known that the healthcare working force entails more women than men, this skewed distribution could have influenced the results. Moreover, the data for this study were obtained in an interview setting and it may therefore be that some respondents did not rank all statements in accordance with their view, or how they actually think and behave in daily practice. Therefore, future studies could consider to complement the data with observation of the behaviours and interactions of professionals and volunteers with patients in relation to the eight domains of PCC. Finally, patients were not included in this study. Patients could share the views presented here, but also have a different view on PCC. Therefore, further research into the views of patients is advocated.

## Conclusion

The most important finding of this study is the difference in opinion when it comes to who is in control of care in the palliative care setting; the patient in the driver’s seat compared to power shared by a team consisting of patients, professionals and volunteers, where the patient is in the passenger seat. Following this line of reasoning there are two views of care, which may lead to different ways of caregiving and support.

## Data Availability

The dataset analyzed during the current study are available from the corresponding author on reasonable request.

## Appendix

**Table 3:** Adaptations made in statements from Berghout et al. (2015) and Cramm et al. (2015)

Original statement	Final statement after adaptation	Why adaptation is made
5. Patients are supported in setting and achieving their own treatment goals.	5. Patients are supported to set and achieve their own goals	In palliative care it is not about ‘treatment goals’ anymore, because patients will not get better. However, they can have other ‘goals’, for example; they still want to tell something to a family member before they die.
New statement	6. Healthcare professionals pay attention to the spiritual and psychosocial needs of patients.	In a pilot interview, there was indicated that spiritual and psychosocial needs of patients are important in the last phase of life.
18. The hospital is accessible for all patients.	19. The building is accessible for all patients.	In this study it is not only about a hospital, but also about a hospice. Therefore we changed this to ‘building’.
19. Clear directions are provided to and inside the hospital.	Removed	This is not very important in the last phase of life, since a hospice is a rather small building.
20. Appointment scheduling is easy.	20. It is easy to schedule a conversation with a doctor or nurse.	Appointment is changed to conversation, since almost all appointments will be a conversation.
21. Waiting times for appointments are acceptable.	21. Waiting times for a request of a patient (for example: a treatment, medication or food) is acceptable	Same as before, appointment is changed in request, since request are more suitable in a palliative care setting than appointments.
22. Language is not a barrier to access to care.	22. Language is not a barrier for access to qualitative good care.	Often patients do have access to care, regardless of their language. However, language could be a barrier for qualitative good care.
New statement	23. Low cognitive functioning (for example: dementia) is not a barrier for receiving good quality of care.	Low cognitive functioning is a very common in the last phase of life and therefore a statement about this is important.
25. Patients receive skilled advice about care and support at home after hospital discharge.	Removed	Patients are not discharged anymore in last phase of life.

## Abbreviations

CCMO: Central Committee on Research Involving Human Subjects
GP: General Practitioner
PCC: Patient-Centred Care
